# Supplementary material for: An improved epigenetic counter to track mitotic age in normal and precancerous tissues
Source: Nat Commun. 2024 May 17;15:4211. doi: 10.1038/s41467-024-48649-8 (PMC11101651; doi:10.1038/s41467-024-48649-8)
Supplement: Supplementary file 3 — Description of Additional Supplementary Files [file 41467_2024_48649_MOESM3_ESM.pdf]

Description of Additional Supplementary Files

File Name: **Supplementary Data 1**
Description: Probe ID, chromosome, position, mapped gene(s) and regulatory region of 371 stemTOC CpGs.

File Name: **Supplementary Data 2**
Description: SampleID, TCGA cancer-type, age, normal/cancer status, mitotic age of the 7 mitotic clocks, and tumor purity estimates from ESTIMATE, ABSOLUTE, LUMP, IHC and CPE for all TCGA samples.

File Name: **Supplementary Data 3**
Description: SampleID, normal tissue-type, age group and mitotic age estimates of 7 mitotic clocks for eGTEX samples.

File Name: **Supplementary Data 4**
Description: Sample ID, cell-type and study, age and mitotic age estimates of 7 mitotic clocks for all sorted immune cells.

File Name: **Supplementary Data 5**
Description: SampleID, EpiSCORE cell-type fraction estimates for TCGA-BLCA samples. EC=endothelial cell, Epi=epithelial cell, Fib=fibroblast, IC=immune-cell.

File Name: **Supplementary Data 6**
Description: SampleID, EpiSCORE cell-type fraction estimates for TCGA-BRCA samples. EC=endothelial, Fib=fibroblast, Lym=lymphocyte, MP=macrophage.

File Name: **Supplementary Data 7**
Description: SampleID, EpiSCORE cell-type fraction estimates for TCGA-CHOL samples. Chol=cholangiocyte, EC=endothelial cell, Hep=hepatocyte, Kup=Kuppfer Macrophage, Lym=lymphocyte.

File Name: **Supplementary Data 8**
Description: SampleID, EpiSCORE cell-type fraction estimates for TCGA-COAD samples. EC=endothelial cell, Epi=epithelial cell, Lym=lymphoid cell, Mye=myeloid cell.

File Name: **Supplementary Data 9**
Description: SampleID, EpiSCORE cell-type fraction estimates for TCGA-ESCA samples.. EC=endothelial cell, Epi=epithelial, Fib=fibroblast, Gland=glandular, IC=immune-cell.

File Name: **Supplementary Data 10**
Description: SampleID, EpiSCORE cell-type fraction estimates for TCGA-GBM samples. Oligo=oligodendrocytes, Astro=astrocytes, OPC=oligodendrocyte progenitor

cells, Endo=endothelial cell

File Name: **Supplementary Data 11**
Description: SampleID, EpiSCORE cell-type fraction estimates for TCGA-HNSC samples. Fib=fibroblast, Gland=glandular, Macro=macrophage, NeuIm=immature neuron, NeuMa= mature neuron, Peri=pericytes.

File Name: **Supplementary Data 12**
Description: SampleID, EpiSCORE cell-type fraction estimates for TCGA-KIRC samples. EC=endothelial cell, Epi=epithelial cell, Fib=fibroblast, IC=immune-cell.

File Name: **Supplementary Data 13**
Description: SampleID, EpiSCORE cell-type fraction estimates for TCGA-KIRP samples. EC=endothelial cell, Epi=epithelial cell, Fib=fibroblast, IC=immune-cell.

File Name: **Supplementary Data 14**
Description: SampleID, EpiSCORE cell-type fraction estimates for TCGA-LGG samples. Oligo=oligodendrocytes, Astro=astrocytes, OPC=oligodendrocyte progenitor cells, Endo=endothelial cell.

File Name: **Supplementary Data 15**
Description: SampleID, EpiSCORE cell-type fraction estimates for TCGA-LIHC samples. Chol=cholangiocyte, EC=endothelial cell, Hep=hepatocyte, Kup=Kuppfer Macrophage, Lym=lymphocyte.

File Name: **Supplementary Data 16**
Description: SampleID, EpiSCORE cell-type fraction estimates for TCGA-LUAD samples. OthEpi=lung epithelial cells which are not alveolar or basal, Lym=lymphocytes, AlvEpi=Alveolar Epithelial, Neu=neutrophils, Neuendocrine=neuroendocrine, Basal=epithelial basal.

File Name: **Supplementary Data 17**
Description: SampleID, EpiSCORE cell-type fraction estimates for TCGA-LUSC samples. OthEpi=lung epithelial cells which are not alveolar or basal, Lym=lymphocytes, AlvEpi=Alveolar Epithelial, Neu=neutrophils, Neuendocrine=neuroendocrine, Basal=epithelial basal.

File Name: **Supplementary Data 18**
Description: SampleID, EpiSCORE cell-type fraction estimates for TCGA-PAAD samples. EC=endothelial cell, IC=immune-cell.

File Name: **Supplementary Data 19**
Description: SampleID, EpiSCORE cell-type fraction estimates for TCGA-PRAD samples. BE=basal epithelial, EC=endothelial cell, Fib=fibroblast, LE=luminal

epithelial, SM=smooth muscle.

File Name: **Supplementary Data 20**
Description: SampleID, EpiSCORE cell-type fraction estimates for TCGA-READ samples. EC=endothelial cell, Epi=epithelial cell, Lym=lymphocyte, Mye=myeloid cell.

File Name: **Supplementary Data 21**
Description: SampleID, EpiSCORE cell-type fraction estimates for TCGA-SKCM samples. EC=endothelial cell, Fib=fibroblast, Kera_diff=differentiated keratinocyte, Kera_undiff=undifferentiated keratinocyte, Macro=macrophage, Mela=melanocyte.
