# Supplementary material for: An improved epigenetic counter to track mitotic age in normal and precancerous tissues
Source: Nat Commun. 2024 May 17;15:4211. doi: 10.1038/s41467-024-48649-8 (PMC11101651; doi:10.1038/s41467-024-48649-8)
Supplement: Supplementary file 5 — Reporting Summary [file 41467_2024_48649_MOESM5_ESM.pdf]

Corresponding author(s): Andrew E Teschendorff

Last updated by author(s): May 3, 2024

Reporting Summary

Nature Portfolio wishes to improve the reproducibility of the work that we publish. This form provides structure for consistency and transparency in reporting. For further information on Nature Portfolio policies, see our Editorial Policies and the Editorial Policy Checklist.

Statistics

For all statistical analyses, confirm that the following items are present in the figure legend, table legend, main text, or Methods section.

n/a	Confirmed	
☐	☒	The exact sample size (*n*) for each experimental group/condition, given as a discrete number and unit of measurement
☐	☒	A statement on whether measurements were taken from distinct samples or whether the same sample was measured repeatedly
☐	☒	The statistical test(s) used AND whether they are one- or two-sided<br>*Only common tests should be described solely by name; describe more complex techniques in the Methods section.*
☐	☒	A description of all covariates tested
☐	☒	A description of any assumptions or corrections, such as tests of normality and adjustment for multiple comparisons
☐	☒	A full description of the statistical parameters including central tendency (e.g. means) or other basic estimates (e.g. regression coefficient) AND variation (e.g. standard deviation) or associated estimates of uncertainty (e.g. confidence intervals)
☐	☒	For null hypothesis testing, the test statistic (e.g. *F*, *t*, *r*) with confidence intervals, effect sizes, degrees of freedom and *P* value noted<br>*Give P values as exact values whenever suitable.*
☒	☐	For Bayesian analysis, information on the choice of priors and Markov chain Monte Carlo settings
☒	☐	For hierarchical and complex designs, identification of the appropriate level for tests and full reporting of outcomes
☐	☒	Estimates of effect sizes (e.g. Cohen's *d*, Pearson's *r*), indicating how they were calculated

Our web collection on statistics for biologists contains articles on many of the points above.

Software and code

Policy information about availability of computer code

Data collection	No software was used to download data.
Data analysis	The following R-packages available from CRAN (www.r-project.org) or Bioconductor (www.bioconductor.org) were used: ggplot2_3.4.2<br>minfi_1.46.0 foreach_1.5.2<br>Biostrings_2.68.1 XVector_0.40.0<br>SummarizedExperiment_1.30.2 Biobase_2.60.0<br>MatrixGenerics_1.12.2 matrixStats_1.0.0<br>GenomicRanges_1.52.0 GenomeInfoDb_1.36.0<br>IRanges_2.34.0 S4Vectors_0.38.1<br>impute_1.74.1 BMIQ_1.6<br>The following R-package EpiSCORE_0.9.5 available from https://github.com/aet21/EpiSCORE was used to estimate cell-type fractions.<br>The following R-package EpiMitClocks_0.1.0 available from https://github.com/aet21/EpiMitClocks was used to estimate mitotic age for all mitotic clocks in this work.<br>A CodeOcean capsule which runs through examples of the EpiMitClocks R-package has been submitted to OceanCode for publication: Available from https://codeocean.com/capsule/3811164/tree

For manuscripts utilizing custom algorithms or software that are central to the research but not yet described in published literature, software must be made available to editors and reviewers. We strongly encourage code deposition in a community repository (e.g. GitHub). See the Nature Portfolio guidelines for submitting code & software for further information.

Data

Policy information about availability of data

All manuscripts must include a data availability statement. This statement should provide the following information, where applicable:

- Accession codes, unique identifiers, or web links for publicly available datasets
- A description of any restrictions on data availability
- For clinical datasets or third party data, please ensure that the statement adheres to our policy

The main Illumina DNA methylation 450k/EPIC datasets used here are freely available from the following public repositories: Endicott (182 cell-line samples, GSE197512 [https://www.ncbi.nlm.nih.gov/geo/query/acc.cgi?acc=GSE197512]); Hannum (656 whole blood, GSE40279, [https://www.ncbi.nlm.nih.gov/geo/query/acc.cgi?acc=GSE40279]) ; MESA (214 purified CD4+ T-cells and 1202 Monocyte samples, GSE56046 [https://www.ncbi.nlm.nih.gov/geo/query/acc.cgi?acc=GSE56046] GSE56581 [https://www.ncbi.nlm.nih.gov/geo/query/acc.cgi?acc=GSE56581]); Tserel (98 CD8+ T-cells, GSE59065 [https://www.ncbi.nlm.nih.gov/geo/query/acc.cgi?acc=GSE59065]); BLUEPRINT (139 matched CD4+ T-cells, Monocytes and Neutrophils, EGAS00001001456 [https://ega-archive.org/studies/EGAS00001001456]) ; Paul (100 B cells, 98 T cells, and 104 monocytes, EGA: EGAS00001001598 [https://ega-archive.org/studies/EGAS00001001598]); Liu (335 whole blood, GSE42861 [https://www.ncbi.nlm.nih.gov/geo/query/acc.cgi?acc=GSE42861]); Pai (n=28 sorted neurons, GSE112179 [https://www.ncbi.nlm.nih.gov/geo/query/acc.cgi?acc=GSE112179]) , Guintivano (n=29 sorted neurons, GSE41826 [https://www.ncbi.nlm.nih.gov/geo/query/acc.cgi?acc=GSE41826]) , Gasparoni (n=16 sorted neurons, GSE66351 [https://www.ncbi.nlm.nih.gov/geo/query/acc.cgi?acc=GSE66351]) and Kozlenkov (n=29 sorted neurons, GSE98203 [https://www.ncbi.nlm.nih.gov/geo/query/acc.cgi?acc=GSE98203]) ; Gastric tissue (191 normal and metaplasia, GSE103186 [https://www.ncbi.nlm.nih.gov/geo/query/acc.cgi?acc=GSE103186]) ; Colon tissue (47 normal and adenoma, E-MTAB-6450 [https://www.ebi.ac.uk/biostudies/arrayexpress/studies/E-MTAB-6450]) 130; Colon tissue2 (123 normal, adenoma, and cancer samples, GSE48684 [https://www.ncbi.nlm.nih.gov/geo/query/acc.cgi?acc=GSE48684]) ; Breast Erlangen (397 normal, GSE69914 [https://www.ncbi.nlm.nih.gov/geo/query/acc.cgi?acc=GSE69914]) ; Breast2 (121 normal, GSE101961 [https://www.ncbi.nlm.nih.gov/geo/query/acc.cgi?acc=GSE101961]) ; Breast Johnson (55 normal and ductal carcinoma in situ samples, GSE66313 [https://www.ncbi.nlm.nih.gov/geo/query/acc.cgi?acc=GSE66313]) ; Liver tissue (137 normal, premalignant and cholangiocarcinoma samples, GSE156299 [https://www.ncbi.nlm.nih.gov/geo/query/acc.cgi?acc=GSE156299]) ; Prostate tissue (36 normal, neoplasia, primary and metastatic samples, GSE116338 [https://www.ncbi.nlm.nih.gov/geo/query/acc.cgi?acc=GSE116338]); Oral tissue (41 normal, lichen planus and oral squamous cell carcinoma samples, GSE123781 [https://www.ncbi.nlm.nih.gov/geo/query/acc.cgi?acc=GSE123781]) ; Esophagus (50 normal, 81 Barrett's Esophagus and 24 adenocarcinomas, GSE104707 [https://www.ncbi.nlm.nih.gov/geo/query/acc.cgi?acc=GSE104707]) Liver-NAFLD (n=325, GSE180474 [https://www.ncbi.nlm.nih.gov/geo/query/acc.cgi?acc=GSE180474]); SCM2 (37 fetal tissue samples, GSE31848 [https://www.ncbi.nlm.nih.gov/geo/query/acc.cgi?acc=GSE31848]) ; Cord-Blood (15 samples, GSE72867 [https://www.ncbi.nlm.nih.gov/geo/query/acc.cgi?acc=GSE72867]); Slieker (34 fetal tissue samples, GSE56515 [https://www.ncbi.nlm.nih.gov/geo/query/acc.cgi?acc=GSE56515]); eGTEX (987 samples from 9 normal tissue-types, GSE213478 [https://www.ncbi.nlm.nih.gov/geo/query/acc.cgi?acc=GSE213478]); Buccal Swabs from Infants (44 samples, GSE229463 [https://www.ncbi.nlm.nih.gov/geo/query/acc.cgi?acc=GSE229463]); Cord Blood from Neonates (128 samples, GSE195595 [https://www.ncbi.nlm.nih.gov/geo/query/acc.cgi?acc=GSE195595]); TCGA data was downloaded from https://gdc.cancer.gov ; The DNAm dataset in buccal cells from the NSHD MRC1946 76 is available by submitting data requests to mrclha.swiftinfo@ucl.ac.uk; see full policy at http://www.nshd.mrc.ac.uk/data.aspx. Managed access is in place for this 69-year-old study to ensure that use of the data is within the bounds of consent given previously by participants and to safeguard any potential threat to anonymity since the participants are all born in the same week. The DNAm-atlas encompassing DNAm reference matrices for 13 tissue-types encompassing over 40 cell-types is freely available from the EpiSCORE R-package https://github.com/aet21/EpiSCORE . Source data are provided with this paper. The remaining data are available within the Article, Supplementary Information or Source Data file.

Research involving human participants, their data, or biological material

Policy information about studies with human participants or human data. See also policy information about sex, gender (identity/presentation), and sexual orientation and race, ethnicity and racism.

Reporting on sex and gender	In this work we only analyzed publicly available data, and sex information is available from the respective publications and data repositories as outlined in the Data availability section. For the mitotic-age analyses considered in this work, sex was not included in study design, although we note that some datasets (e.g. normal breast and breast cancer tissue) were composed entirely of women. Reason sex was not considered in the reported study design is that mitotic age results are not found to be influenced by sex. Hence, to maximize power it is more reasonable to present all the results for men and women together.
Reporting on race, ethnicity, or other socially relevant groupings	As we only analyzed publicly available data, information on ethnicity if available is provided in the respective publications. For the analyses considered in this work, ethnicity was not found to be a confounder, except in the Hannum et al cohort, where it highly correlated with a technical batch effect (plate), and so for this dataset adjustment for ethnicity was done via an adjustment for plate.
Population characteristics	NA (only publicly available data were analyzed and this information was already provided in the respective publications).
Recruitment	NA (only publicly available data were analyzed and this information was already provided in the respective publications).
Ethics oversight	NA (only publicly available data were analyzed and this information was already provided in the respective publications).

Note that full information on the approval of the study protocol must also be provided in the manuscript.

Field-specific reporting

Please select the one below that is the best fit for your research. If you are not sure, read the appropriate sections before making your selection.

☒ Life sciences ☐ Behavioural & social sciences ☐ Ecological, evolutionary & environmental sciences

For a reference copy of the document with all sections, see nature.com/documents/nr-reporting-summary-flat.pdf

Life sciences study design

All studies must disclose on these points even when the disclosure is negative.

Sample size	The only part of the MS where this is relevant is in the construction of stemTOC where we used 3 large whole blood cohorts (each around 700 samples big). Using 3 large cohorts gave us ample power to confidently identify a significant number of age-DMCs to allow for the construction of stemTOC.
Data exclusions	The only excluded data was 1 cell-line from the data of Endicott et al. One cell-line (AG21839), which displayed global non-monotonic DNAm patterns with population doublings (PDs) was removed, as the non-monotonicity raises concerns about quality.
Replication	The correlation of stemTOC's mitotic with tumor cell of origin is demonstrated across 15 independent TCGA cancer-types. The increased mitotic age of stemTOC in precancerous lesion was demonstrated across 9 different tissues. All attempts at replication were successful.
Randomization	This study only analyzes publicly available data, as such no new data is generated. Randomization refers to the need to perform such procedure in the context of generating new data where all potential confounders should be randomized relative to the outcome.
Blinding	This study only analyzes publicly available data, as such no new data is generated. Hence blinding is not relevant to this study.

Reporting for specific materials, systems and methods

We require information from authors about some types of materials, experimental systems and methods used in many studies. Here, indicate whether each material, system or method listed is relevant to your study. If you are not sure if a list item applies to your research, read the appropriate section before selecting a response.

Materials & experimental systems

n/a	Involved in the study
☒	☐ Antibodies
☒	☐ Eukaryotic cell lines
☒	☐ Palaeontology and archaeology
☒	☐ Animals and other organisms
☒	☐ Clinical data
☒	☐ Dual use research of concern
☒	☐ Plants

Methods

n/a	Involved in the study
☒	☐ ChIP-seq
☒	☐ Flow cytometry
☒	☐ MRI-based neuroimaging
